# Associations of oxidative stress markers with the prevalence of sarcopenia in the United States general population

**DOI:** 10.1016/j.clinsp.2024.100450

**Published:** 2024-08-02

**Authors:** Tingting Sang, Feng Gao, Xiao Lu, Ying Yang, Lingling Liu, Gang Zhang, Guosong Han

**Affiliations:** a Department of Rehabilitation, The People's Hospital of Suzhou New District, Suzhou, Jiangsu, China; b Department of Orthopedics, Kunshan Hospital of Traditional Chinese Medicine, Kunshan, Jiangsu, China; c Department of Rehabilitation, The First Affiliated Hospital of Nanjing Medical University, Nanjing, Nanjing, Jiangsu, China; d Department of Rehabilitation, The First Affiliated Hospital of Anhui Medical University, Shushan District, Hefei, Anhui, China; e Department of Orthopaedics, The First People's Hospital of Hefei City, Hefei, Anhui, China

**Keywords:** American population, Cross-sectional study, Markers of oxidative stress, Sarcopenia

## Abstract

**Objective:**

The purpose of the present study was to examine the association of oxidative stress markers with sarcopenia in the general United States population under the age of 60.

**Methods:**

We used the National Health and Nutrition Examination Survey data from 2011‒2014 and performed Restricted Cubic Spline (RCS) plots, weighted multivariable logistic regression analysis to calculate ratio ratios and 95% Confidence Intervals, and subgroup analysis based on age, sex, hypertension, diabetes mellitus, and body mass index stratification to determine the association of markers of oxidative stress with the prevalence of sarcopenia.

**Results:**

The present analysis included a total of 8,782 participants. Firstly, the RCS plots showed a roughly L-shaped curve association of total bilirubin and serum iron with a prevalence of sarcopenia. Secondly, albumin was negatively and linearly associated with the risk of sarcopenia. Finally, with the increase in gamma-glutamyl transferase, the prevalence of sarcopenia showed a trend of first rising and then declining as a result of the iron increase.

**Conclusions:**

We demonstrated a nonlinear association between markers of oxidative stress and sarcopenia. The need to focus more on levels of oxidative stress in the body could provide better prevention strategies for sarcopenia.

## Highlights

• A nonlinear association between markers of oxidative stress and sarcopenia.
• Alb was negatively correlated with the prevalence of sarcopenia.
• The roughly L-curve association of total bilirubin and serum iron with sarcopenia.
• Maintaining levels of oxidative stress markers is critical to preventing sarcopenia.

## Introduction

Sarcopenia is a condition characterized by a gradual decline in muscle mass, strength, and physical performance that increases as individuals age.^1^ Sarcopenia occurs mainly in older adults because from approximately the fifth decade of age, the annual loss of muscle mass is 0.8% and the loss of strength is 3%.^2^^,^^3^ The current estimated global prevalence of sarcopenia among older adults (> 60 years) ranges between 10% and 16%.^4^ Among community dwellers aged 60 years and older, the estimated prevalence of sarcopenia is approximately 11% in men and 9% in women.^5^ However, among hospital inpatients, the rates are considerably higher, with upwards of one in every four adult men and women having this condition. Nursing home residents have shown even higher rates, with a prevalence estimate of this condition affecting one-half of elderly men and one-third of elderly women.^6^

A multitude of well-established risk factors contribute to the development of sarcopenia, including advancing age, sedentary behavior, inadequate nutritional intake, the presence of chronic diseases, and age-related hormonal alterations.^7^^,^^8^ Early identification and intervention are paramount in mitigating the progression of sarcopenia; however, the intricate and multifaceted nature of its underlying mechanisms complicates early detection efforts.^9^^,^^10^ Various biological pathways have been proposed to elucidate the intricate interplay between oxidative stress and sarcopenia, thereby offering potential avenues for early detection through the evaluation of oxidative stress markers.^7^^,^^8^^,^^11^ Oxidative stress markers such as Glutathione (GSH) and Glutathione Disulfide (GSSG) function as antioxidants, safeguarding cells against oxidative damage, and imbalances in the ratio of reduced glutathione to oxidized glutathione disulfide have been associated with sarcopenia.^7^ In addition to the above markers of oxidative stress, there are some common markers of oxidative stress in hematology tests. Bilirubin is a powerful antioxidant whose elevation correlates directly with lipid prooxidant activity.^12^ As a negative acute-phase reactant, Albumin (Alb) also has antioxidative properties extracellularly.^13^ It has been shown that serum Gamma-Glutamyl Transferase (GGT), a marker of oxidative stress and responsible for extracellular glutathione catabolism, may play a role in cardiovascular disease, peripheral arterial disease, and hypertension.^14^ The micronutrient iron is essential to human health. Iron, however, can also function as a pro-oxidant transition metal due to its redox properties. Overproduction of reactive oxygen species and oxidative stress can result from excessive amounts of iron, which is also associated with a higher risk of cardiovascular disease and type 2 diabetes.^15^ These markers are routinely assessed in blood or urine specimens and offer valuable insights into the oxidative stress status of individuals afflicted with sarcopenia.^9^ While it is imperative to recognize that no individual marker comprehensively encapsulates the complexity of these processes, a thorough understanding of the association of each marker with sarcopenia is essential for establishing a robust foundation for the utilization of a combination of markers to augment risk assessment and facilitate disease progression monitoring.^11^^,^^16^ Moreover, the prevalence of sarcopenia may vary by region due to differences in demographics, lifestyle factors, and healthcare access.^17^ Regions with rapidly aging populations, such as East Asia and Europe, may have higher rates of sarcopenia.^17^^,^^18^ Additionally, socioeconomic factors can influence the prevalence of sarcopenia, with disparities observed between developed and developing countries. Understanding the role of oxidative stress in sarcopenia in specific regions may provide avenues for developing tailored detection and treatment strategies. Therefore, in this study, we will utilize the National Health and Nutrition Examination Survey (NHANES) database to explore associations of oxidative stress markers with the risk of sarcopenia, in order to advance understanding of the complex interactions in U.S. based context.

## Material and methods

### Study population

**Fig. 1:**
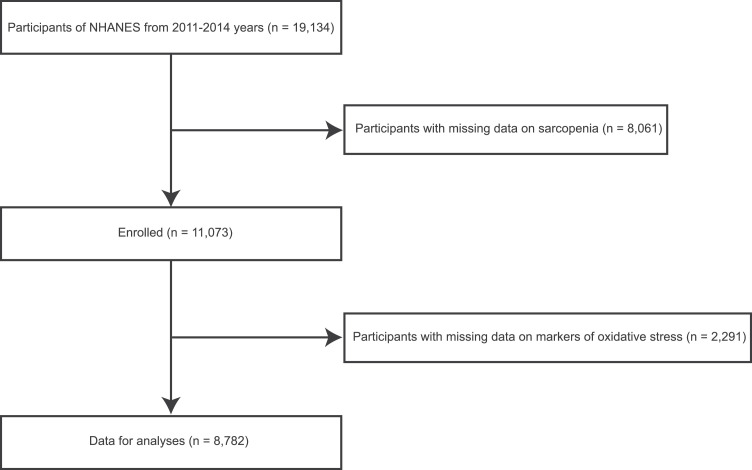
Study flow chart. NHANES, National Health and Nutrition Examination Surveys.

The NHANES database is an ongoing U.S. national population-based nutrition and health survey. It uses complex, multi-stage, and probability sampling techniques rather than a simple random sample based on the U.S. population.^19^ Of the 19,134 participants initially included in the present study, 8,061 were excluded because of missing data on sarcopenia. 2,291 were excluded because of missing data on measures of oxidative stress. Finally, 8,782 participants were included and analyzed (Fig. 1). The National Center for Health Statistics (NCHS) Research Ethics Review Board approved the study. All participants provided written informed consent when recruited.^20^ More information about the data can be found at (https://www.cdc.gov/nchs/nhanes/index.htm). The study was an observational study (cross-sectional studies) and followed the STROBE Statement.

### Covariates

All the following covariates in the present study include demographic data, test results, survey results, dietary data, and lab results. Age, family Poverty Income Ratio (PIR), race/ethnicity, marital status, sex, and education level were all included in the demographic data. Body Mass Index (BMI), and waist circumference were examined data. Questionnaire results included information on smoking status and drinking status. Dietary data included mean energy intake. Finally, estimated Glomerular Filtration Rate (eGFR), Fast Glucose (FBG), Triglyceride (TG), Total Cholesterol (TC), High-Density Lipoprotein-Cholesterol (HDL-C), serum Uric Acid (sUA), Serum creatinine (Scr), total bilirubin, Alb, Blood Urea Nitrogen (BUN), GGT, and serum iron were measured in the laboratory.

### Measurement of oxidative stress markers

Serum specimens are processed, stored (-30°C), and shipped to the National Center for Environmental Health for testing. An in-depth description of how to collect and process instructions are provided in the NHANES Laboratory/Medical Technologists Procedures Manual. Professional technicians operated and used the UniCel DxC 800 Synchron Clinical System (Beckman Coulter, Brea, California) and the Beckman Coulter UniCel DxC 660i Synchron Access chemistry analyzers to measure the results of total bilirubin, Alb, and GGT.^21^ Additionally, the serum iron concentration was measured using the DCX-800 system.^22^

### The sarcopenia measurement

Sarcopenia was diagnosed using a Dual-energy X-Ray Absorptiometry (DXA) scan. The DXA scan provides a precise measurement of muscle mass and strength, making it an accurate method for diagnosing sarcopenia. Appendicular Lean Mass (ALM) was defined as the sum of the fat-free masses of all four extremities (arms and legs). For this study, Foundation for the National Institutes of Health criteria was used for ALM-defined sarcopenia (< 19.75 kg in males, < 15.02 kg in females) and ALM adjusted for Body Mass Index (BMI) (< 0.789 kg for males, < 0.512 kg for females).^23^^,^^24^

### Statistical analysis

In the study, all statistical analyses were performed using R version 4.2.3 (R Foundation for Statistical Computing, Vienna, Austria), and SPSS version 22.0 (SPSS Inc., Chicago, IL, USA).

There was statistical significance at the p-value <0.05. Makers of oxidative stress were all divided into quartiles, including total bilirubin (Q1: 0.10‒0.50, Q2: 0.51‒0.60, Q3: 0.61‒0.80, and Q4: 0.81‒7.10), Alb (Q1: 24.0‒41.0, Q2: 41.1‒44.0, Q3: 44.1‒46.0, and Q4: 46.1‒56.0), GGT (Q1: 4.0‒12.0, Q2: 12.1‒17.0, Q3: 17.1‒25.0, and Q4: 25.1‒1510.0), and serum iron (Q1: 5.0‒59.0, Q2: 59.1‒80.0, Q3: 80.1‒105.0, and Q4: 105.1‒557.0). The mean ± standard deviation was used to express continuous variables, and categorical variables were expressed as frequencies and percentages. The weighted Student's *t*-test and weighted Chi-Square test were performed to compare the continuous variables, and constituent ratios between each group, respectively. The Restricted Cubic Spline (RCS) plot and weighted multivariate logistic regression analysis were performed to explore the potential nonlinearity of the association of markers of inflammation and oxidative stress with the prevalence of sarcopenia. A total of three models were constructed for adjustment (Model 1, Model 2, and Model 3). Firstly, Model 1 was adjusted for age and sex. Second, Model 2 was further adjusted for marital status, smoking status, education level, family PIR, race/ethnicity, the complication of hypertension, and DM, and drinking status. Finally, Model 3 was further adjusted for BMI, waist circumference, mean energy intake, FBG, UA, TC, Scr, TG, HDL-C, BUN, and eGFR. Additionally, subgroup analysis stratified by age, sex, hypertension, and DM was applied to examine the link between measures of inflammation, and oxidative stress and the prevalence of sarcopenia.

## Results

### Characteristics of participants

The characteristics of the study population are shown in Table 1. This research comprised 8,782 participants overall. Of these, 145 individuals had sarcopenia, and 1.7% of the total population. Sex, education level, the complication of hypertension, and DM, drinking status, waist circumference, BMI, UA, eGFR, total bilirubin, HDL, Alb, serum iron, FBG, and TG had significant differences among non-sarcopenia and sarcopenia groups.

**Table 1:** Demographic characteristics of the study participants.

Variables	Overall (n = 8,782)	Non-sarcopenia (n = 8,637)	Sarcopenia (n = 145)	p-value
Age, years	35.62 ± 0.31	35.60 ± 0.33	36.68 ± 1.98	0.615
Sex (%)				<0.001
Male	4363 (49.7%)	4251 (48.4%)	112 (1.3%)	
Female	4419 (50.3%)	4386 (49.9%)	33 (0.7%)	
Race (%)				0.159
Mexican American	1300 (14.8%)	1264 (14.4%)	36 (0.4%)	
Other Hispanic	852 (9.7%)	841 (9.6%)	11 (0.1%)	
Non-Hispanic Black	2024 (23.0%)	1999 (22.8%)	25 (0.3%)	
Non-Hispanic White	3082 (35.1%)	3027 (34.5%)	55 (0.6%)	
Other race				
Family PIR	2.81 ± 0.08	2.82 ± 0.08	2.56 ± 0.15	0.099
Education level (%)				0.009
Less than high school	2993 (34.1%)	2913 (33.2%)	80 (0.9%)	
High school	1643 (18.7%)	1621 (18.5%)	22 (0.3%)	
More than high school	4146 (47.2%)	4103 (46.7%)	43 (0.5%)	
Marital status (%)				0.206
Having a partner	4453 (50.7%)	4396 (50.1%)	57 (0.6%)	
No partner	979 (11.1%)	961 (10.9%)	18 (0.2%)	
Unmarried	3350 (38.1%)	3280 (37.3%)	70 (0.8%)	
Hypertension (%)				<0.001
No	6862 (78.1%)	6782 (77.2%)	80 (0.9%)	
Yes	1920 (21.9%)	1885 (21.1%)	65 (0.7%)	
DM (%)				<0.001
No	8044 (91.6%)	7932 (90.3%)	112 (1.3%)	
Yes	738 (8.4%)	705 (8.0%)	33 (0.4%)	
Smoker (%)				0.885
No	5627 (64.1%)	5536 (63.0%)	91 (1.0%)	
Former	1201 (13.7%)	1177 (13.4%)	24 (0.3%)	
Now	1954 (22.3%)	1924 (21.9%)	30 (0.3%)	
Alcohol user (%)				<0.001
No	1878 (21.4%)	1827 (20.8%)	51 (0.6%)	
Former	875 (10.0%)	853 (9.7%)	22 (0.3%)	
Mild	2336 (26.6%)	2309 (26.3%)	27 (0.3%)	
Moderate	1419 (16.2%)	1414 (16.1%)	5 (0.1%)	
Heavy	2274 (25.9%)	2234 (25.4%)	40 (0.5%)	
BMI, kg/m^2^	28.10 ± 0.17	27.89 ± 0.16	40.31 ± 1.20	<0.001
Waist circumference, cm	95.66 ± 0.39	95.20 ± 0.38	121.92 ± 2.48	<0.001
Mean energy	2159.43 ± 11.78	2160.06 ± 11.51	2123.05 ± 76.08	0.617
intake (kcal/day)				
Total bilirubin, g/dL	0.68 ± 0.01	0.68 ± 0.01	0.60 ± 0.03	0.009
Alb, g/L	43.51 ± 0.08	43.53 ± 0.08	42.18 ± 0.37	<0.001
GGT, iu/L	25.58 ± 0.56	25.52 ± 0.57	29.16 ± 2.09	0.106
Serum iron, ug/mL	86.59 ± 0.67	86.85 ± 0.66	71.45 ± 4.23	0.001
FBG, mg/dL	100.30 ± 0.37	99.92 ± 0.38	122.43 ± 8.84	0.017
TC, mg/dL	186.72 ± 0.80	186.82 ± 0.84	181.30 ± 5.65	0.359
TG, mg/dL	121.45 ± 1.76	120.86 ± 1.75	155.54 ± 5.91	<0.001
HDL, mg/dL	52.08 ± 0.30	52.26 ± 0.30	41.85 ± 0.91	<0.001
BUN, mg/dL	11.78 ± 0.07	11.77 ± 0.07	12.61 ± 0.58	0.160
UA, mg/dL	5.29 ± 0.03	5.28 ± 0.03	6.05 ± 0.19	<0.001
Scr, mg/dL	0.84 ± 0.00	0.84 ± 0.00	0.80 ± 0.03	0.159
eGFR, mL/min/1.73 m^2^	106.34 ± 0.42	106.20 ± 0.44	114.62 ± 3.57	0.032

Family PIR, Poverty Income Ratio; DM, Diabetes Mellitus; BMI, Body Mass Index; FBG, Fast Glucose; Alb, Albumin; GGT, Gamma Glutamyl Transferase; TC, Total Cholesterol; TG, Triglycerides; HDL-cholesterol, High Density Lipoprotein-cholesterol; BUN, Blood Urea Nitrogen; UA, Uric Acid; Scr, Serum Creatinine; eGFR, Estimated Glomerular Filtration Rate.

### Associations of oxidative stress markers with sarcopenia

**Fig. 2:**
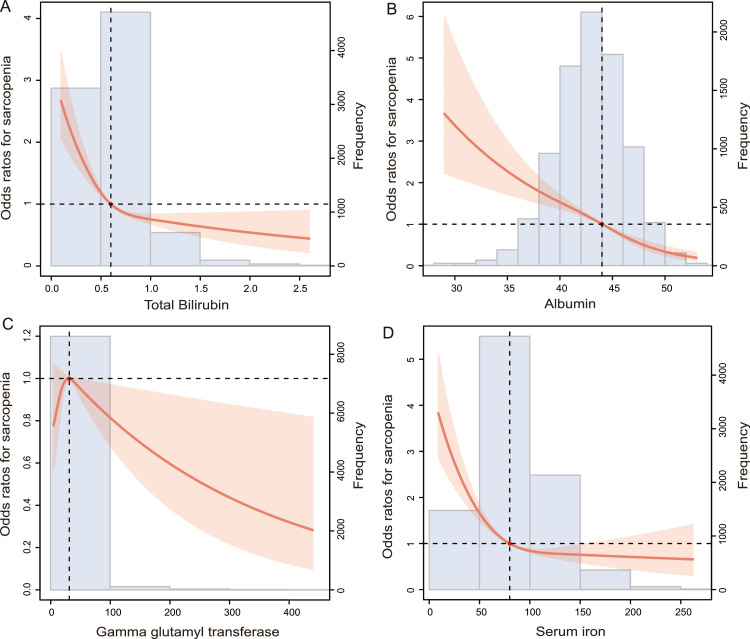
The restricted cubic spline plot of the association of (A) total bilirubin (B) albumin (C) gamma glutamyl transferase and (D) serum iron with prevalence of sarcopenia. RCS, Restricted Cubic Spline.

The RCS plot is shown in Fig. 2A and D, representing a roughly L-shaped curve association of total bilirubin and serum iron with the prevalence of sarcopenia (p for nonlinearity = 0.148 and 0.098). Additionally, Alb had a negative and linear correlation with the prevalence of sarcopenia (Fig. 2B; p for nonlinearity = 0.363). As GGT increased, the risk of sarcopenia was shown to display a trend of first rising and then declining (Fig. 2C; p for nonlinearity = 0.037). Finally, the authors have presented the results of the multivariate logistic regression analysis of markers of oxidative stress and sarcopenia in Table 2.

**Table 2:** Associations of markers of oxidative stress with prevalence of sarcopenia.

	Model 1	p for trend	Model 2	p for trend	Model 3	p for trend
	OR (95%CI)		OR (95%CI)		OR (95%CI)	
**Total bilirubin**		<0.001		<0.001		0.032
0.10‒0.50	1.00		1.00		1.00	
0.51‒0.60	0.38 (0.23, 0.64)^c^		0.40 (0.24, 0.67)^c^		0.63 (0.36, 1.11)	
0.61‒0.80	0.30 (0.19, 0.48)^c^		0.33 (0.21, 0.53)^c^		0.57 (0.34, 0.96)^a^	
0.81‒7.10	0.29 (0.17, 0.47)^c^		0.33 (0.20, 0.55)^c^		0.57 (0.33, 1.01)	
**Alb**		<0.001		<0.001		0.003
24.0‒41.0	1.00		1.00		1.00	
41.1‒44.0	0.40 (0.27, 0.59)^c^		0.43 (0.29, 0.65)^c^		0.67 (0.41, 1.08)	
44.1‒46.0	0.15 (0.08, 0.26)^c^		0.16 (0.08, 0.29)^c^		0.39 (0.18, 0.86)	
46.1‒56.0	0.14 (0.07, 0.25)^c^		0.13 (0.07, 0.25)^c^		0.32 (0.16, 0.66)	
**GGT**		<0.001		<0.001		0.733
4.0‒12.0	1.00		1.00		1.00	
12.1‒17.0	3.27 (1.85, 5.76)^c^		3.15 (1.71, 5.78)^c^		1.10 (0.57, 2.13)	
17.1‒25.0	2.44 (1.37, 4.33)^b^		2.87 (1.58, 5.22)^c^		1.09 (0.55, 2.16)	
25.1‒1510.0	1.50 (0.83, 2.71)		1.67 (0.92, 3.05)		0.97 (0.50, 1.85)	
**Serum iron**		<0.001		<0.001		0.001
5.0‒59.0	1.00		1.00		1.00	
59.1‒80.0	0.59 (0.40, 0.88)^a^		0.60 (0.40, 0.90)^a^		0.92 (0.58, 1.46)	
80.1‒105.0	0.26 (0.16, 0.42)^c^		0.26 (0.16, 0.44)^c^		0.45 (0.25, 0.79)^a^	
105.1‒557.0	0.18 (0.11, 0.32)^c^		0.19 (0.11, 0.33)^c^		0.45 (0.24, 0.86)^a^	

a p < 0.05;

b p < 0.01;

c p < 0.001. Alb, Albumin; GGT, Gamma Glutamyl Transferase; OR, Odd Ratio; CI, Confidence Interval. Model 1: Age and sex. Model 2: Model 1 variables plus race/ethnicity, education level, marital status, family poverty-income ratio, the complication of hypertension, and diabetes mellitus, smoker, and alcohol user; Model 3 was adjusted for Model 2 variables plus body mass index, waist circumference, mean energy intake, fast glucose, total cholesterol, and triglyceride, high-density lipoprotein-cholesterol, blood urea nitrogen, serum uric acid, serum creatinine, and estimated glomerular filtration rate.

### Subgroup analysis

A subgroup analysis was conducted to further investigate the association of markers of oxidative stress with the prevalence of sarcopenia, stratified by age, sex, hypertension and DM (Supplementary Figs. 1‒4; Supplementary Tables 1‒4). The roughly L-shaped curve association of total bilirubin with sarcopenia was found among participants under the age of 45, who were female, with or without hypertension (Supplementary Fig. 1). There was a linear negative correlation between albumin and sarcopenia in the under 45 years, male, with or without hypertension and without DM population (Supplementary Fig. 2). Additionally, the authors also found that the roughly L-shaped curve association of total bilirubin with sarcopenia existed in participants under the age of 45, with or without hypertension and without DM (Supplementary Fig. 4).

## Discussion

In this study, the authors observed negative linear correlations of Alb with the prevalence of sarcopenia in the U.S. population, in which individuals exhibiting higher levels of Alb may be indicative of a decreased susceptibility to developing sarcopenia. Furthermore, there was a roughly L-shaped correlation between total bilirubin as well as serum iron and sarcopenia risk. Finally, the analysis revealed the trend of first rising and then falling between GGT and the prevalence of sarcopenia. The maintenance of optimal levels of inflammation and oxidative stress markers within the body is paramount in the prevention of sarcopenia. According to a review of studies, Alb was negatively associated with frailty and sarcopenia regardless of the participant's age and setting.^25^ Due to its association with malnutrition, Alb is a nutritional indicator of body dysfunction. As a result, this decrease in albumin in old age is highly associated with sarcopenia risk. In this study, the results are consistent. Additionally, a study by Uemura K et al. also found that sarcopenia and low serum albumin levels synergistically increased disabled incidence in older adults.^26^ Therefore, for people with sarcopenia who are at risk of malnutrition, appropriate nutritional interventions should be taken. In the British population, Petermann-Rocha F et al. found that subjects with sarcopenia had higher levels of GGT than those without sarcopenia.^27^ Studies have shown a correlation between GGT and insulin resistance or chronic inflammation as metabolic risk factors for sarcopenia. Therefore, higher levels of GGT may indicate sarcopenia.^28^ The end product of haem metabolism, bilirubin, has been shown to have antioxidative properties.^29^ Wang C et al. revealed that men, but not women, showed a positive association between total bilirubin and appendicular skeletal muscle mass index.^30^ This finding is in line with the results of the study. Finally, iron plays an important role in the function of erythrocytes, oxidative stress, and immune response in the body. Poor physical performance has been associated with low iron blood serum concentrations, according to Beard JL.^31^ Additionally, Additionally, Xu B et al. reported that individuals with sarcopenia had lower serum iron levels than those without sarcopenia.^32^ This is consistent with the present results. On the other hand, Ho V and Nakagawa C have shown a significant association between serum ferritin and transferrin saturation and reduced grip strength, but not serum iron.^33^^,^^34^ Further, Sha T and his colleagues found that a one SD increment in genetically determined serum iron levels correlated with a 53% increase in sarcopenia risk.^35^ It may be due to the differences in study design and outcome measures that there is this inconsistency.

By utilizing NHANES, which offers a large, nationally representative sample, the authors are able to conduct a thorough examination of these associations across diverse demographic groups. This enhances the generalizability of the present findings to the broader U.S. population. The utilization of standardized procedures and protocols for data collection within NHANES further strengthens the consistency and reliability of the present study's findings over various survey cycles. This allows for better validity, comparability, and facilitates the replication of this study. However, it is important to acknowledge certain limitations associated with the NHANES database. The primarily cross-sectional design of NHANES prevents us from establishing causal relationships, limiting our ability to infer the temporal sequences of events and determine the directionality of the observed associations.^36^ Additionally, the reliance on self-reported data for certain variables introduces the possibility of recall bias or social desirability bias, potentially influencing the accuracy and reliability of the collected information.^37^ Despite these limitations, the comprehensive nature of the NHANES database and its large sample size provide a valuable platform for investigating the associations between oxidative stress markers and sarcopenia risk. This contributes to a deeper understanding of the intricate interplay between biological mechanisms and musculoskeletal health within the U.S. population.

## Conclusion

1. In the U.S. general population, Alb was negatively correlated with the prevalence of sarcopenia. Individuals with higher levels of Alb could be considered as people with a lower risk of developing sarcopenia.
2. Additionally, the authors also found a roughly L-curve association of total bilirubin and serum iron with sarcopenia risk. Maintaining the levels of oxidative stress markers in the body is crucial to the prevention of sarcopenia.
3. These findings provide a foundational framework for future investigations exploring the dynamics of oxidative stress markers and their underlying mechanistic implications in the onset and progression of sarcopenia.

## Ethical approval and consent to participate

All NHANES participants provided written informed consent and the National Center for Health Statistics obtained institutional review board approval prior to data collection (Protocol #2011-17). And the study was approved by the ethics committee for medical research at the People's Hospital of Suzhou New District (2024-022).

## Funding

This work was supported by the National Natural Science Foundation of China (82072546), the Research Fund of Anhui Institute of Translational Medicine (2022zhyx-C90), and the Special funding project of Basic and Clinical Cooperative Research Promotion Plan of the Third Affiliated Hospital of Anhui Medical University (2023sfy015).

## CRediT authorship contribution statement

**Tingting Sang:** Data curation, Investigation, Resources, Writing – original draft, Writing – review & editing. **Feng Gao:** Formal analysis, Methodology. **Xiao Lu:** Software, Validation. **Ying Yang:** Software, Validation. **Lingling Liu:** Software, Validation. **Gang Zhang:** Conceptualization, Methodology, Software, Writing – original draft, Writing – review & editing. **Guosong Han:** Conceptualization, Methodology, Software, Writing – original draft, Writing – review & editing.

## Declaration of competing interest

The authors declare no conflicts of interest.

## Appendix Supplementary materials

Image, application 1
