# Supplementary material for: Associations of oxidative stress markers with the prevalence of sarcopenia in the United States general population
Source: Clinics (Sao Paulo). 2024 Aug 2;79:100450. doi: 10.1016/j.clinsp.2024.100450 (PMC11334777; doi:10.1016/j.clinsp.2024.100450)
Supplement: Supplementary file 1 [file mmc1.docx]

**CLINICS-D-24-00272_ Supplementary Material**

**Supplementary Table 1** Subgroups analysis of total bilirubin with the prevalence of sarcopenia.

| **Total bilirubin** | **Q1** | **Q2** | **Q3** | **Q4** | **p for trend** | **p for interaction** |
| --- | --- | --- | --- | --- | --- | --- |
|  | **OR (95%CI)** | **OR (95%CI)** | **OR (95%CI)** | **OR (95%CI)** |  |  |
| Age |  |  |  |  |  | 0.073 |
| < 40 | Ref. | 0.68 (0.30, 1.51) | 0.54 (0.26, 1.15) | 0.34 (0.13, 0.91)^a^ | 0.016 |  |
| ≥ 40 | Ref. | 0.79 (0.33, 1.90) | 0.91 (0.40, 2.11) | 1.51 (0.65, 3.49) | 0.495 |  |
| Sex |  |  |  |  |  | 0.025 |
| Male | Ref. | 0.64 (0.34, 1.23) | 0.63 (0.33, 1.19) | 0.43 (0.22, 0.80)^a^ | 0.027 |  |
| Female | Ref. | 0.41 (0.11, 1.50) | 0.68 (0.12, 3.93) | 1.36 (0.52, 3.60) | 0.965 |  |
| Hypertension |  |  |  |  |  | 0.170 |
| No | Ref. | 0.76 (0.34, 1.70) | 0.53 (0.25, 1.12) | 0.52 (0.22, 1.24) | 0.227 |  |
| Yes | Ref. | 0.87 (0.39, 1.94) | 0.83 (0.37, 1.86) | 0.75 (0.29, 1.91) | 0.502 |  |
| Diabetes mellitus |  |  |  |  |  | 0.003 |
| No | Ref. | 0.38 (0.19, 0.77)^a^ | 0.43 (0.23, 0.80)^a^ | 0.68 (0.36, 1.29) | 0.037 |  |
| Yes | Ref. | 3.81 (1.03, 14.03)^a^ | 2.41 (0.62, 9.34) | 1.63 (0.30, 8.88) | 0.307 |  |

Abbreviations: Q1: 68.67‒314.40; Q2: 314.41‒426.44; Q3: 426.45‒618.4; Q4: 618.42‒2635.00; ^a^ p < 0.05

OR, Odd Ratio; CI, Confidence Interval. Analysis was adjusted for age, sex, race/ethnicity, education level, marital status, family poverty-income ratio, the complication of hypertension, and diabetes mellitus, smoker, alcohol user, body mass index, waist circumference, mean energy intake, fast glucose, total cholesterol, and triglyceride, high-density lipoprotein-cholesterol, blood urea nitrogen, serum uric acid, serum creatinine, and estimated glomerular filtration rate.

**Supplementary Table 2** Subgroups analysis of Alb with the prevalence of sarcopenia.

| **Alb** | **Q1** | **Q2** | **Q3** | **Q4** | **p for trend** | **p for interaction** |
| --- | --- | --- | --- | --- | --- | --- |
|  | **OR (95%CI)** | **OR (95%CI)** | **OR (95%CI)** | **OR (95%CI)** |  |  |
| Age |  |  |  |  |  | 0.014 |
| < 40 | Ref. | 0.70 (0.33, 1.50) | 0.37 (0.13, 1.07) | 0.17 (0.06, 0.50) | 0.009 |  |
| ≥ 40 | Ref. | 0.61 (0.07, 5.23) | 1.12 (0.52, 2.40) | 1.69 (0.56, 5.15) | 0.774 |  |
| Sex |  |  |  |  |  | 0.049 |
| Male | Ref. | 0.59 (0.33, 1.06) | 0.25 (0.10, 0.64^a^ | 0.21 (0.09, 0.49)^b^ | <0.001 |  |
| Female | Ref. | 0.96 (0.33, 2.83) | 2.06 (0.46, 9.27) | 5.63 (0.96, 33.20) | 0.113 |  |
| Hypertension |  |  |  |  |  | 0.110 |
| No | Ref. | 0.34 (0.19, 0.61) | 0.09 (0.04, 0.21) | 0.09 (0.04, 0.19) | <0.001 |  |
| Yes | Ref. | 0.96 (0.23, 4.03) | 0.89 (0.43, 1.84) | 0.85 (0.27, 2.63) | 0.831 |  |
| Diabetes mellitus |  |  |  |  |  | 0.130 |
| No | Ref. | 0.60 (0.34, 1.06) | 0.36 (0.15, 0.86) | 0.28 (0.13, 0.61) | 0.004 |  |
| Yes | Ref. | 2.19 (0.66, 7.29) | 0.75 (0.06, 9.11) | 0.63 (0.04, 8.45) | 0.963 |  |

Abbreviations: Q1: 27‒40; Q2: 41‒42; Q3: 43‒44; Q4: 45‒51 ^a^ p < 0.01; ^b^ p < 0.001.

OR, Odd Ratio; CI, Confidence Interval; Alb, albumin. Analysis was adjusted for age, sex, race/ethnicity, education level, marital status, family poverty-income ratio, the complication of hypertension, and diabetes mellitus, smoker, alcohol user, body mass index, waist circumference, mean energy intake, fast glucose, total cholesterol, and triglyceride, high-density lipoprotein-cholesterol, blood urea nitrogen, serum uric acid, serum creatinine, and estimated glomerular filtration rate.

**Supplementary Table 3** Subgroups analysis of GGT with the prevalence of sarcopenia.

| **GGT** | **Q1** | **Q2** | **Q3** | **Q4** | **p for trend** | **p for interaction** |
| --- | --- | --- | --- | --- | --- | --- |
|  | **OR (95%CI)** | **OR (95%CI)** | **OR (95%CI)** | **OR (95%CI)** |  |  |
| Age |  |  |  |  |  | 0.025 |
| < 40 | Ref. | 0.58 (0.20, 1.69) | 0.98 (0.44, 2.19) | 1.02 (0.41, 2.52) | 0.376 |  |
| ≥ 40 | Ref. | 6.32 (1.23, 32.55)^a^ | 3.48 (0.66, 18.34) | 2.61 (0.44, 15.27) | 0.005 |  |
| Sex |  |  |  |  |  | 0.004 |
| Male | Ref. | 0.85 (0.38, 1.91) | 0.81 (0.37, 1.76) | 0.76 (0.32, 1.80) | 0.599 |  |
| Female | Ref. | 2.40 (0.58, 9.88) | 1.97 (0.48, 8.03) | 1.67 (0.40, 7.03) | 0.219 |  |
| Hypertension |  |  |  |  |  | 0.285 |
| No | Ref. | 1.69 (0.87, 3.31) | 3.00 (1.47, 6.13)^b^ | 5.82 (2.81, 12.03)^c^ | <0.001 |  |
| Yes | Ref. | 3.37 (0.57, 20.14) | 4.40 (0.81, 23.76) | 3.45 (0.64, 18.78) | 0.376 |  |
| Diabetes mellitus |  |  |  |  |  | 0.085 |
| No | Ref. | 1.20 (0.57, 2.52) | 1.15 (0.52, 2.58) | 1.11 (0.54, 2.28) | 0.733 |  |
| Yes | Ref. | 1.60 (0.20, 12.47) | 1.16 (0.19, 7.24) | 0.32 (0.03, 12.47) | 0.450 |  |

Abbreviations: Q1: 5‒15; Q2: 16‒20; Q3: 21‒29; Q4: 30‒462; ^a^p<0.05; ^b^p<0.01; ^c^p<0.001.

GGT, Gamma Glutamyl Transferase; OR, Odd Ratio; CI, Confidence Interval. Analysis was adjusted for age, sex, race/ethnicity, education level, marital status, family poverty-income ratio, the complication of hypertension, and diabetes mellitus, smoker, alcohol user, body mass index, waist circumference, mean energy intake, fast glucose, total cholesterol, and triglyceride, high-density lipoprotein-cholesterol, blood urea nitrogen, serum uric acid, serum creatinine, and estimated glomerular filtration rate.

**Supplementary Table 4** Subgroups analysis of serum iron with the prevalence of sarcopenia.

| **Serum iron** | **Q1** | **Q2** | **Q3** | **Q4** | **p for trend** | **p for interaction** |
| --- | --- | --- | --- | --- | --- | --- |
|  | **OR (95% CI)** | **OR (95% CI)** | **OR (95% CI)** | **OR (95% CI)** |  |  |
| Age |  |  |  |  |  | 0.373 |
| < 40 | Ref. | 0.90 (0.41, 1.87) | 0.42 (0.18, 1.01) | 0.43 (0.17, 1.05) | 0.017 |  |
| ≥ 40 | Ref. | 1.33 (0.62, 2.84) | 1.15 (0.48, 2.73) | 1.04 (0.38, 2.83) | 0.928 |  |
| Sex |  |  |  |  |  | 0.001 |
| Male | Ref. | 0.72 (0.41, 1.27) | 0.29 (0.14, 0.60)^c^ | 0.36 (0.18,0.73)^b^ | <0.001 |  |
| Female | Ref. | 2.33 (0.82, 6.60) | 1.67 (0.65, 4.27) | 0.94 (0.18, 4.92) | 0.373 |  |
| Hypertension |  |  |  |  |  | 0.851 |
| No | Ref. | 0.52 (0.30, 0.90)^a^ | 0.21 (0.10, 0.43)^c^ | 0.16 (0.08, 0.34)^c^ | <0.001 |  |
| Yes | Ref. | 0.97 (0.41, 2.46) | 0.72 (0.25, 2.07) | 0.67 (0.28, 1.62) | 0.313 |  |
| Diabetes mellitus |  |  |  |  |  | 0.389 |
| No | Ref. | 0.89 (0.52, 1.54) | 0.46 (0.23, 0.91)^a^ | 0.40 (0.20, 0.79)^a^ | 0.004 |  |
| Yes | Ref. | 0.97 (0.25, 3.68) | 0.91 (0.30, 2.79) | 0.27 (0.03, 2.42) | 0.422 |  |

Abbreviations: Q1: 9‒66; Q2: 67‒84; Q3: 85‒106; Q4: 107‒283; ^a^p<0.05; ^b^p<0.01; ^c^p<0.001.

OR, Odd Ratio; CI, Confidence Interval. Analysis was adjusted for age, sex, race/ethnicity, education level, marital status, family poverty-income ratio, the complication of hypertension, and diabetes mellitus, smoker, alcohol user, body mass index, waist circumference, mean energy intake, fast glucose, total cholesterol, and triglyceride, high-density lipoprotein-cholesterol, blood urea nitrogen, serum uric acid, serum creatinine, and estimated glomerular filtration rate.

**Supplementary Figure 1** Subgroup analysis for the relationship between total bilirubin and sarcopenia based on (A) age, (B) sex, (C) hypertension, and (D) DM. DM, Diabetes Mellitus.

**Supplementary Figure 2** Subgroup analysis for the relationship between Alb and sarcopenia based on (A) age, (B) sex, (C) hypertension, and (D) DM. Alb, Albumin; DM, Diabetes Mellitus.

**Supplementary Figure 3** Subgroup analysis for the relationship between GGT and sarcopenia based on (A) age, (B) sex, (C) hypertension, and (D) DM. GGT, gamma glutamyl transferase; DM, Diabetes Mellitus.

**Supplementary Figure 4** Subgroup analysis for the relationship between serum iron and sarcopenia based on (A) age, (B) sex, (C) hypertension, and (D) DM. DM, Diabetes Mellitus.
